# Supplementary material for: Applicability of Pre-Plastic Deformation Method for Improving Mechanical Properties of Bulk Metallic Glasses
Source: Materials (Basel). 2022 Oct 28;15(21):7574. doi: 10.3390/ma15217574 (PMC9654271; doi:10.3390/ma15217574)
Supplement: Supplementary file 1 [file materials-15-07574-s001.zip › materials-1937154-supplementary.pdf]

Supplementary Materials

Applicability of Pre-Plastic Deformation Method for Improving Mechanical Properties of Bulk Metallic Glasses

Changshan Zhou [1,2], Hezhi Zhang [1,2], Xudong Yuan [1,3,*], Kaikai Song [1,2,*] and Dan Liu [4]

[1] Shenzhen Research Institute of Shandong University, Shenzhen 518057, China
[2] School of Mechanical, Electrical and Information Engineering, Shandong University, Weihai 264209, China
[3] Department of Materials Science, University of Leoben, A-8700 Leoben, Austria
[4] Weihai Wanfeng Magnesium Industry Science and Technology Development Co., Ltd., Weihai 264209, China;
* Correspondence: xudong.yuan@oeaw.ac.at (X.Y.); songkaikai8297@gmail.com (K.S.); Tel.: +86-06315688338 (K.S.)

Supplementary files:

Figure S1. EDS spectrum of the as-cast $Zr_{56}Co_{28}Al_{16}$ BMG measured at random regions.

Figure S2. EDS spectrum of the as-cast $Zr_{46}Cu_{46}Al_{8}$ BMG measured at random regions.

Figure S3. EDS spectrum of the as-cast $Fe_{48}Cr_{15}Mo_{14}Y_{2}C_{15}B_{6}$ BMG measured at random regions.

Table S1. The STZ volumes and Poisson's ratios of reported BMGs.

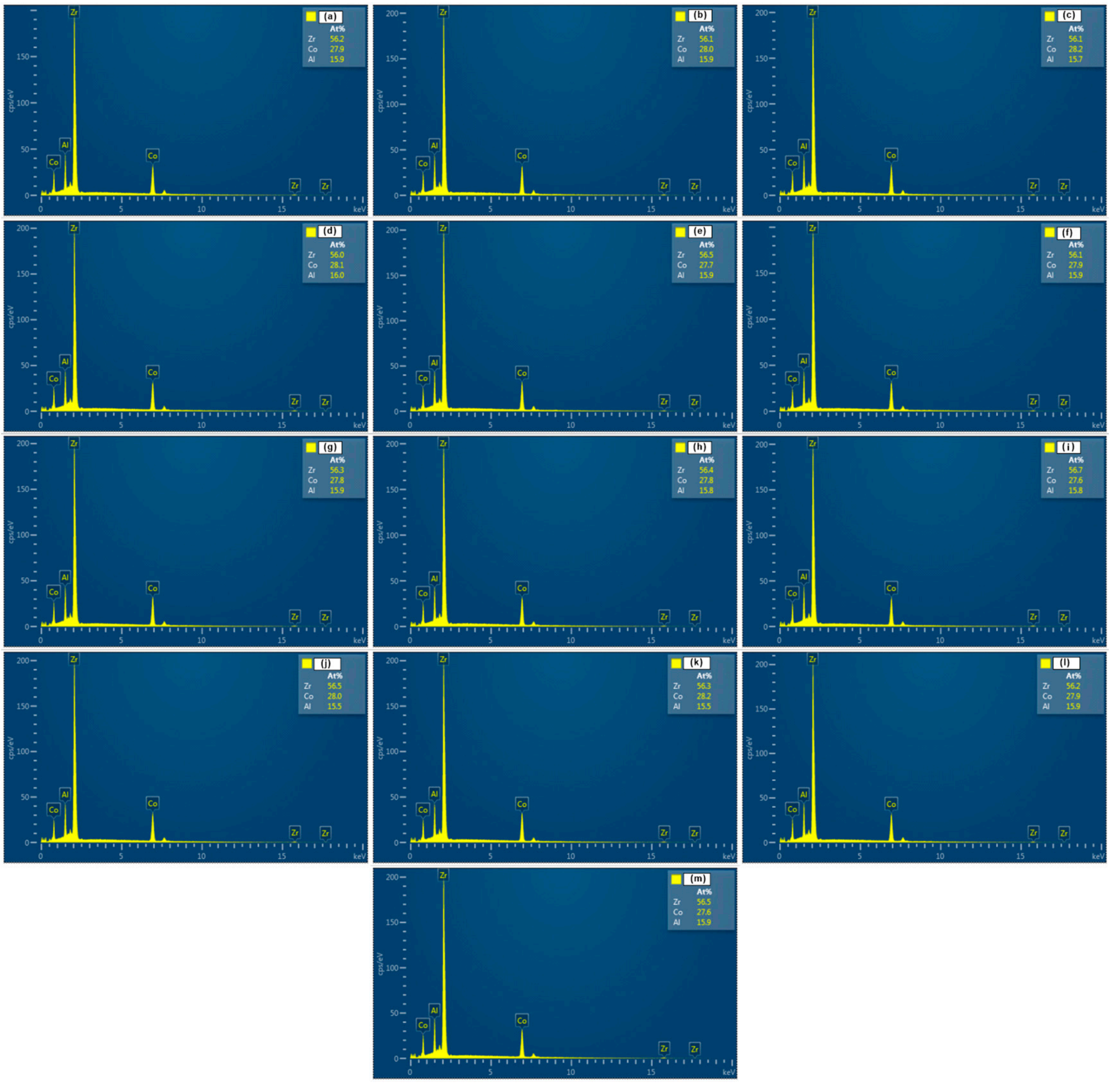

Figure S1. EDS spectrum of the as-cast $Zr_{56}Co_{28}Al_{16}$ BMG measured at random regions.

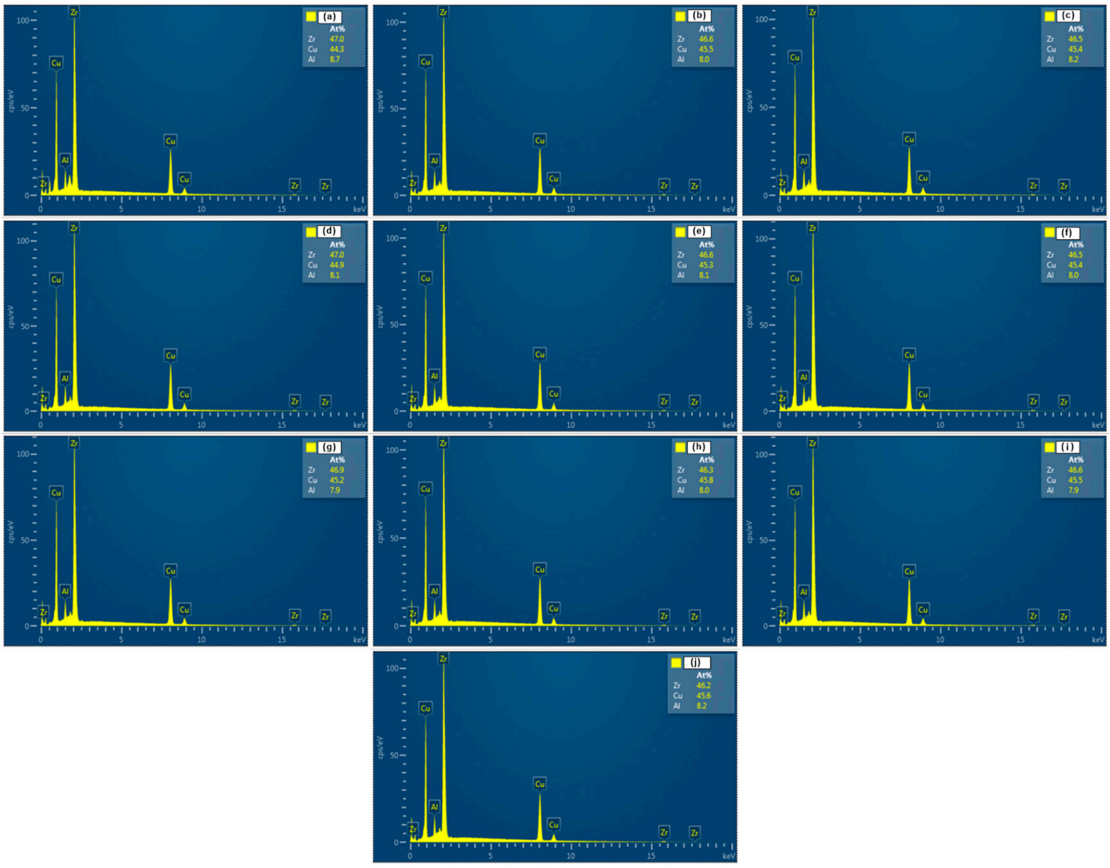

Figure S2. EDS spectrum of the as-cast $Zr_{46}Cu_{46}Al_8$ BMG measured at random regions

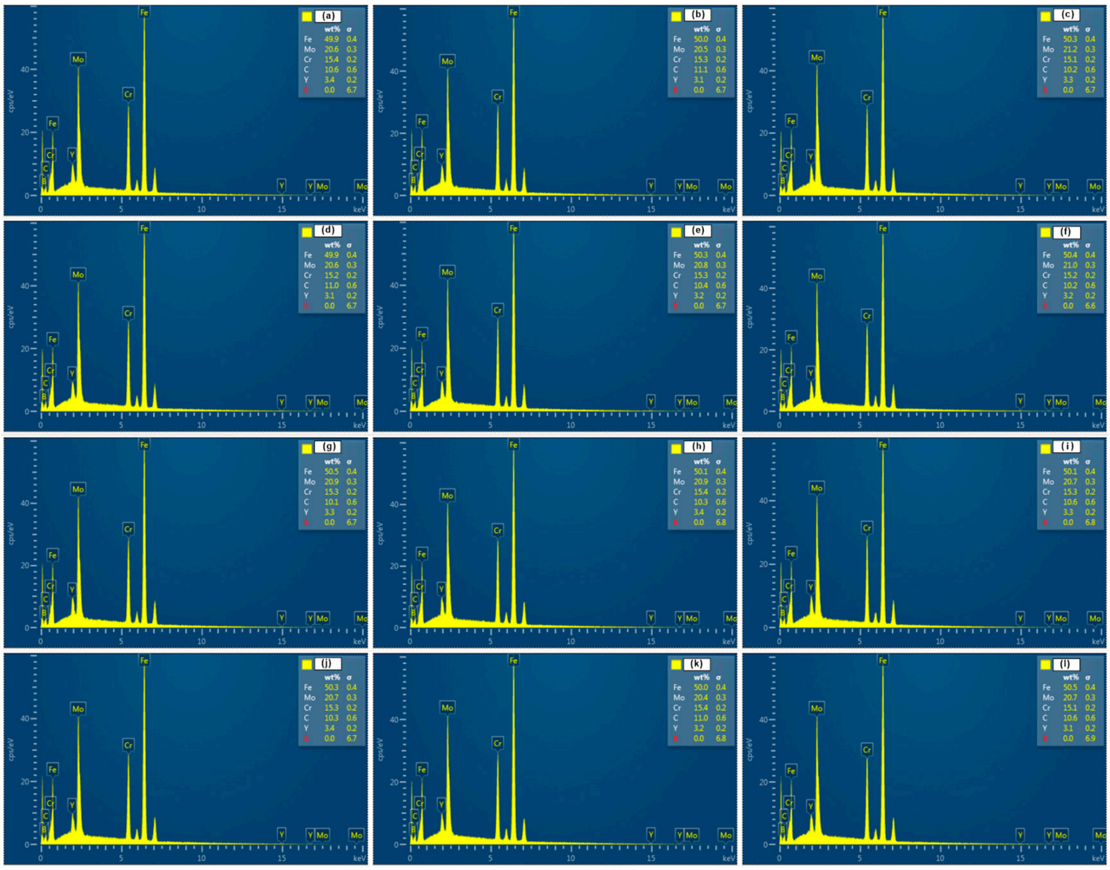

Figure S3 EDS spectrum of the as-cast $Fe_{48}Cr_{15}Mo_{14}Y_{2}C_{15}B_{6}$ BMGmeasured at random regions.

Table S1: The STZ volumes and Poisson's ratios of reported BMGs.

Compositions	STZ volume (nm^3)	Poisson's Ratio	Refs.
Zr55Cu30Ni5Al10	3.04	-	[1]
Zr55Cu30Ni5Al10	3.94	-	[1]
Zr55Cu30Ni5Al10	4.16	-	[1]
Zr55Cu25Ni10Al10	3.89	-	[1]
Zr46Cu30.14Ag8.36Al8Be7.5	3.38	-	[1]
Zr53.7Cu28.5Ni9.4Al8.4	4.18	-	[1]
Pd40Ni10Cu30P20	3.5	0.399	[1]
La50Al35Ni15	5.27	0.328	[2]
La55Al25Ni20	5.31	0.326	[2]
La60Al25Ni15	5.5	0.325	[2]
La70Al15Ni15	5.03	0.324	[2]
La55Al15Ni10Cu10Co15	5.37	0.342	[2]
La57.5Cu12.5Ni12Al18	6.13	0.33	[2]
La57.5(Cu50Ni50)25Al17.5	6.18	0.348	[2]
Zr46.75Ti8.25Cu7.5Ni10Be27.5	3.13	0.355	[2]
Zr57Cu15.4Ni12.6Al10Nb5	3.95	0.365	[2]
Zr53.7Cu28.5Ni9.4Al8.4	4.18	0.372	[2]
Zr64.13Cu15.75Ni10.12Al10	3.63	0.377	[2]
Zr65Cu15Ni10Al10	4.24	0.377	[2]
Pd40Ni10Cu30P20	3.5	0.399	[2]
Pd43Ni10Cu27P20	2.8	0.404	[2]
Pd48Ni32P20	2.36	0.4	[2]

Cu45Zr45Al10	3.95	0.364	[2]
Cu50Zr50)92Al8	4.75	0.366	[2]
Cu47Zr47Al6	4.29	0.367	[2]
Tm39Y16Al25Co20	4.76	0.304	[2]
Al82Ni10Ce8	5.71	0.324	[2]
Ni45Nb30Zr25	0.33	-	[3]
Ni45Nb30Zr25	0.318	-	
Ni33Nb22Zr40Co5	0.465	-	[3]
Ni33Nb22Zr40Co5	0.465	-	[3]
Ni27Nb18Zr50Co5	0.343	-	[3]
Ni27Nb18Zr50Co5	0.514	-	[3]
Ni35Nb30Zr15Ti10Fe5Co5	0.272	-	[3]
Ni35Nb30Zr15Ti10Fe5Co5	0.219	-	[3]
Zr52.5Cu17.9Ni14.6Al10Ti5Vit 105	0.347	0.373	[4]
Zr52.5Cu17.9Ni14.6Al10Ti5Vit 105	0.335	0.373	[4]
Zr52.5Cu17.9Ni14.6Al10Ti5Vit 105	0.337	0.373	[4]
Zr52.5Cu17.9Ni14.6Al10Ti5Vit 105	0.464	-	[4]
Zr52.5Cu17.9Ni14.6Al10Ti5Vit 105	0.443	-	[4]
Zr55Cu25Ni10Al10	3.89	0.33	[5]
Zr44Cu44Al6Ag6	2.54	0.32	[5]
Zr55Cu30Al10Ni5	3.429	0.378	[5]
Zr70Ni16Cu6Al8	15.7	0.393	[5]
Zr45Cu49Al6	1.73	0.365	[5]
Zr54Cu38Al8	2.95	0.366	[5]

Zr46.75Ti8.25Cu7.5Ni10Be27.5	3.13	0.355	[5]
Zr57Cu15.4Ni12.6Al10Nb5	3.95	0.365	[5]
Zr53.7Cu28.5Ni9.4Al8.4	4.18	0.372	[5]
Zr64.13Cu15.75Ni10.12Al10	3.63	0.377	[5]
Zr65Cu15Ni10Al10	4.24	0.377	[5]
Zr50.7Cu28Ni9Al12.3	6.01	0.37	[5]
Zr52.5Cu17.9Ni14.6Al10Ti5	2.94	0.37	[5]
Zr54.65Al11.5Ni4.43Cu27.49Y1.93 As-cast	1.22	0.353	[5]
Zr54.65Al11.5Ni4.43Cu27.49Y1.93-DCT20	1.9	0.366	[5]
Zr54.65Al11.5Ni4.43Cu27.49Y1.93-DCT40	2.31	0.369	[5]
Zr54.65Al11.5Ni4.43Cu27.49Y1.93-DCT60	2.83	0.374	[5]
La50Al35Ni15	5.27	0.328	[5]
La55Al25Ni20	5.31	0.326	[5]
La60Al25Ni15	5.5	0.325	[5]
La70Al15Ni15	5.03	0.324	[5]
La55Al15Ni10Cu10Co15	5.37	0.342	[5]
La57.5Cu12.5Ni12Al18	6.13	0.33	[5]
La57.5Cu12.5Ni12.5Al17.5	6.18	0.348	[5]
Cu45Zr45Al10	3.95	0.364	[5]
Cu46Zr46Al8	4.75	0.366	[5]
Cu47Zr47Al6	4.29	0.367	[5]
Cu60Hf25Ti15	4.3	0.38	[5]
Pd40Ni40P20	6.56	0.41	[5]
Pd40Ni10Cu30P20	3.5	0.399	[5]

Pd43Ni10Cu27P20	2.8	0.404	[5]
Pd48Ni32P20	2.36	0.4	[5]
Pt57.5Cu14.7Ni5.3P22.5	5.95	0.39,0.42	[5,6]
Ni53Nb20Ti10Zr8Co6Cu3	2.81	0.33	[5]
Tm39Y16Al25Co20	4.76	0.304	[5]
Al82Ni10Ce8	5.71	0.324	[5]
Zr50Ti5Cu27Ni10Al8	1.975	-	[7]
Zr50Ti5Cu27Ni10Al8	2.181	-	[7]
Zr50Ti5Cu27Ni10Al8	2.119	-	[7]
Zr50Ti5Cu27Ni10Al8	2.099	-	[7]
Zr50Ti5Cu27Ni10Al8	1.781	-	[7]
Ti16.7Zr16.7Hf16.7Cu16.7Ni16.7Be16.7	0.383	-	[8]
Ti16.7Zr16.7Hf16.7Cu16.7Ni16.7Be16.7	0.364	-	[8]
Ti16.7Zr16.7Hf16.7Cu16.7Ni16.7Be16.7	0.319	-	[8]
Ti16.7Zr16.7Hf16.7Cu16.7Ni16.7Be16.7	0.304	-	[8]
Ti16.7Zr16.7Hf16.7Cu16.7Ni16.7Be16.7	0.291	-	[8]
Zr64·13Cu15.75Ni10·12Al10	1.58	0.377	[9]
Zr46(Cu4.5/5.5Ag1/5.5)46Al8	1.42	0.364	[9]
Cu46Zr46Al8	1.75	0.363	[9]
La65Al14(Cu5/6Ag1/6)11(Ni1/2Co1/2)10	2.06	0.353	[9]
La66Al14Cu10Ni10	1.94	0.336	[9]
La55Al25Ni5Cu10Co5	1.82	0.341	[9]
Fe68Mo4Cr1Y5B22	0.56	0.277	[9]
Co39·78B26.2Ni21·42Si7.8Ta4.8	0.67	0.334	[9]

Cu48Zr48Al4	0.27222	0.37	[10]
Cu48Zr48Al4	0.21288	-	[10]
Cu48Zr48Al4	0.22764	-	[10]
Cu48Zr48Al4	0.243	-	[10]
Au50Cu25.5Si17Ag7.5	1.24	-	[11]
Au60Cu15.5Si17Ag7.5	0.58	-	[11]
Au65Cu10.5Si17Ag7.5	0.59	-	[11]
Ti33Zr30Cu9Ni5.5Be22.5	5.76	-	[12]
Ti33Zr30Cu9Ni5.5Be22.5	7.3	-	[12]
Ti33Zr30Cu9Ni5.5Be22.5	9.07	-	[12]
Ti33Zr30Cu9Ni5.5Be22.5	9.94	-	[12]
Ti33Zr30Cu9Ni5.5Be22.5	6.44	-	[12]
Zr50.7Cu28Ni9Al12.3	6.01	0.37	[13]
Zr50.7Cu28Ni9Al12.3	3.27	0.365	[13]
Zr50.7Cu28Ni9Al12.3	17.92	0.413	[13]
Ti20Zr20Hf20Be20Cu20	1.82	0.348	[14,15]
Ti20Zr20Hf20Be20Cu20	1.96	-	[14]
Ti20Zr20Hf20Be20Cu20	2.29	-	[14]
Ti20Zr20Hf20Be20Cu20	2.74	-	[14]
Ti20Zr20Hf20Be20Cu20	3.44	-	[14]
Cu48Zr48Al4	12.15	0.37	[16]
Cu47.5Zr47.5Al5	8.27	0.372	[16]
Cu47Zr47Al6	5.99	0.367	[16]
Cu46.5Zr46.5Al7	5.52	-	[16]

Cu46Zr46Al8	4.82	0.366	[16]
Cu45Zr46Al8Co1	4.5	-	[16]
Cu44Zr46Al8Co2	5.51	-	[16]
Zr46Cu30.14Ag8.36Al8Be7.5	3.38	0.357	[17]
Zr46Cu30.14Ag8.36Al8Be7.5)70(Zr75Cu25)30	4.53	0.373	[17]
Zr46Cu30.14Ag8.36Al8Be7.5)70(Zr75Cu25)30-620K	6.42	0.362	[17]
La60Ni25Al15	4.3	-	[18]
La60Ni25Al15	4.86	-	[18]
La60Ni25Al15	3.85	-	[18]
Zr52.5Cu17.9Ni14.6Al10Ti5 (Vit 105)	0.387	0.373	[19]
Zr52.5Cu17.9Ni14.6Al10Ti5 (Vit 105)	0.337	0.373	[19]
Zr52.5Cu17.9Ni14.6Al10Ti5 (Vit 105)	0.314	0.373	[19]
Zr52.5Cu17.9Ni14.6Al10Ti5 (Vit 105)	0.229	0.373	[19]
Zr52.5Cu17.9Ni14.6Al10Ti5 (Vit 105)	0.226	0.373	[19]
Zr70Ni16Cu6Al8	15.7	0.384	[20,21]
Zr70Ni16Cu6Al8	10.65	-	[20]
Zr70Ni16Cu6Al8	7.6	-	[20]
La60Ni15Al25	2.644	-	[22]
La60Ni15Al25	2.466	-	[22]
La60Ni15Al25	2.344	-	[22]
La60Ni15Al25	1.844	-	[22]
Ti36.1Zr33.2Ni5.8Be24.9)76Cu24	0.77	-	[23]
Ti36.1Zr33.2Ni5.8Be24.9)76Cu24	1.84	-	[23]
Ti36.1Zr33.2Ni5.8Be24.9)76Cu24	5.83	-	[23]

Ti36.1Zr33.2Ni5.8Be24.9)76Cu24	2.9	-	[23]
Ti36.1Zr33.2Ni5.8Be24.9)76Cu24	1.6	-	[23]
Ti36.1Zr33.2Ni5.8Be24.9)76Cu24	0.94	-	[23]
Ti20Zr20Hf20Be20Cu20	1.907	0.348	[24]
Ti20Zr20Hf20Be20Cu7.5Ni12.5	1.552	0.354	[24]
Ti20Zr20Hf20Be20Ni20	1.16	0.353	[24]
Zr55Cu30Al10Ni5	2.278	0.378	[25]
Zr55Cu30Al10Ni5	2.473	-	[25]
Zr55Cu30Al10Ni5	3.362	-	[25]
Zr35Ti30Cu8.25Be26.75	0.296816	-	[26]
Zr35Ti30Cu8.25Be26.75	0.30164	-	[26]
Zr35Ti30Cu8.25Be26.75	0.323999	-	[26]
Zr35Ti30Cu8.25Be26.75	0.333166	-	[26]

References

1. Dong, F.; He, M.; Zhang, Y.; Wang, B.; Luo, L.; Su, Y.; Yang, H.; Yuan, X., Investigation of shear transformation zone and ductility of Zr-based bulk metallic glass after plasma-assisted hydrogenation. *Mater. Sci. Eng., A* **2019,** 759, 105-111.
2. Liu, S.T.; Wang, Z.; Peng, H.L.; Yu, H.B.; Wang, W.H., The activation energy and volume of flow units of metallic glasses. *Scripta Mater.* **2012,** 67, 9-12.
3. Zhao, Y.; Choi, I.C.; Seok, M.Y.; Kim, M.H.; Kim, D.H.; Ramamurty, U.; Suh, J.Y.; Jang, J.I., Effect of hydrogen on the yielding behavior and shear transformation zone volume in metallic glass ribbons. *Acta Mater.* **2014,** 78, 213-221.
4. Choi, I.C.; Zhao, Y.; Kim, Y.J.; Yoo, B.G.; Suh, J.Y.; Ramamurty, U.; Jang, J.I., Indentation size effect and shear transformation zone size in a bulk metallic glass in two different structural states. *Acta Mater.* **2012,** 60, (19), 6862-6868.
5. Zhu, Q.; Zhang, M.; Jin, X.; Yang, H.; Jia, L.; Qiao, J., Effect of deep cryogenic cycling treatment on shear transformation zone volume and size of Zr-based metallic glass. *J. Mater. Res.* **2021,** 36, 2047-2055.
6. Lewandowski , J.J.; Wang, W.H.; Greer, A.L., Intrinsic plasticity or brittleness of metallic glasses. *Philos. Mag. Lett.* **2005,** 85, 77-87.
7. Liu, S.; Chang, Z.; Fu, Y.; Liu, Y.; Lin, M.; Ren, X.; Wang, W.; Zhang, Z.; He, J., Nanoscale creep behavior and its size dependency of a Zr-based bulk metallic glass manufactured by selective laser

melting. *Mater. Des.* **2022,** 218, 110723.
8. Tong, Y.; Qiao, J.C.; Pelletier, J.M.; Yao, Y., Rate-dependent plastic deformation of TiZrHfCuNiBe high entropy bulk metallic glass. *J. Alloys Compd.* **2019,** 785, 542-552.
9. Zhao, X.N.; Cao, Q.P.; Wang, C.; Wang, X.D.; Zhang, D.X.; Qu, S.X.; Jiang, J.Z., Dependence of room-temperature nanoindentation creep behavior and shear transformation zone on the glass transition temperature in bulk metallic glasses. *J. Non-Cryst. Solids* **2016,** 445-446, 19-29.
10. Fan, H.; Wang, N.; He, C.; Huang, Y.; Ning, Z.; Sun, J., Effect of pre-straining on the structure and nano-mechanical properties of a CuZrAl bulk metallic glass. *J. Alloys Compd.* **2022,** 918, 165635.
11. Pan, D.; Guo, H.; Zhang, W.; Inoue, A.; Chen, M.W., Temperature-induced anomalous brittle-to-ductile transition of bulk metallic glasses. *Proc. Natl. Acad. Sci. U.S.A.* **2011,** 99, 241907.
12. Lv, J.W.; Wang, F.L.; Yin, D.W.; Zhang, S.; Cai, Z. Q.; Shi, Z.L.; Ma, M.Z.; Zhang, X.Y., Effect of deep cryogenic cycling treatment on the microstructure and mechanical properties of Ti-based bulk metallic glass. *J. Alloys Compd.* **2021,** 887, 161386.
13. Chen, Z.Q.; Huang, L.; Huang, P.; Xu, K.W.; Wang, F.; Lu, T.J., Clarification on shear transformation zone size and its correlation with plasticity for Zr-based bulk metallic glass in different structural states. *Mater. Sci. Eng., A* **2016,** 677, 349-355.
14. Gong, P.; Yin, G.; Jamili-Shirvan, Z.; Ding, H.; Wang, X.; Jin, J., Influence of deep cryogenic cycling on the rejuvenation and plasticization of TiZrHfBeCu high-entropy bulk metallic glass. *Mater. Sci. Eng., A* **2020,** 797, 140078.
15. Zhao, S.F.; Yang, G.N.; Ding, H.Y.; Yao, K.F., A quinary Ti-Zr-Hf-Be-Cu high entropy bulk metallic glass with a critical size of 12 mm. *Intermetallics* **2015,** 61, 47-50.
16. Limbach, R.; Kosiba, K.; Pauly, S.; Kühn, U.; Wondraczek, L., Serrated flow of CuZr-based bulk metallic glasses probed by nanoindentation: Role of the activation barrier, size and distribution of shear transformation zones. *J. Non-Cryst. Solids* **2017,** 459, 130-141.
17. Cao, Q.P.; Jin, J.B.; Ma, Y.; Cao, X.Z.; Wang, B.Y.; Qu, S.X.; Wang, X.D.; Zhang, D.X.; Jiang, J.Z., Enhanced plasticity in Zr-Cu-Ag-Al-Be bulk metallic glasses. *J. Non-Cryst. Solids* **2015,** 412, 35-44.
18. Liu, Z.; Huang, P.; Wang, F., The correlation between β relaxation and shear transformation zone in LaNiAl bulk metallic glasses: The effect of cryogenic thermal cycling treatment. *J. Alloys Compd.* **2021,** 865, 158993.
19. Choi, I.C.; Zhao, Y.; Yoo, B.G.; Kim, Y.J.; Suh, J.Y.; Ramamurty, U.; Jang, J.I., Estimation of the shear transformation zone size in a bulk metallic glass through statistical analysis of the first pop-in stresses during spherical nanoindentation. *Scripta Mater.* **2012,** 66, 923-926.
20. Pan, D.; Yokoyama, Y.; Fujita, T.; Liu, Y.H.; Kohara, S.; Inoue, A.; Chen, M.W., Correlation between structural relaxation and shear transformation zone volume of a bulk metallic glass. *Appl. Phys. Lett.* **2009,** 95, 141909.
21. Li, Y.H.; Zhang, W.; Dong, C.; Qiang, J.B.; Yubuta, K.; Makino, A.; Inoue, A., Unusual compressive plasticity of a centimeter-diameter Zr-based bulk metallic glass with high Zr content. *J. Alloys Compd.* **2010,** 504, S2-S5.
22. Ma, Z.; Huang, P.; Wang, F., Effects of cryogenic thermal cycling on a La-based metallic glass: Relaxation or rejuvenation? *J. Alloys Compd.* **2022,** 909, 164741.
23. Khanouki, M.T.A.; Tavakoli, R.; Aashuri, H., Temperature dependence of serrated flow, strain-rate sensitivity and activation volume in a Ti-based bulk metallic glass. *J. Non-Cryst. Solids* **2021,**

553, 120497.
24. Zhao, S.; Wang, H.; Gu, J.; Guo, N.; Shao, L.; Zhang, Y.; Yao, K.; Chen, N., High strain rate sensitivity of hardness in Ti-Zr-Hf-Be-(Cu/Ni) high entropy bulk metallic glasses. *J. Alloys Compd.* **2018,** 742, 312-317.
25. Dong, F.; Chu, Y.; He, M.; Zhang, Y.; Li, W.; Liaw, P.K.; Wang, B.; Luo, L.; Su, Y.; Ritchie, R.O.; Yuan, X., Manipulating internal flow units toward favorable plasticity in Zr-based bulk-metallic glasses by hydrogenation. *J. Mater. Sci. Technol.* **2022,** 102, 36-45.
26. Saini, P.; Narayan, R.L., On simultaneous enhancement in local yield strength and plasticity of short-term annealed bulk metallic glasses. *J. Alloys Compd.* **2022,** 898, 162960.
